# Synthesis and Different Effects of Biotinylated PAMAM G3 Dendrimer Substituted with Nimesulide in Human Normal Fibroblasts and Squamous Carcinoma Cells

**DOI:** 10.3390/biom9090437

**Published:** 2019-09-01

**Authors:** Łukasz Uram, Aleksandra Filipowicz-Rachwał, Maria Misiorek, Aleksandra Winiarz, Elżbieta Wałajtys-Rode, Stanisław Wołowiec

**Affiliations:** 1 Faculty of Chemistry, Rzeszow University of Technology, 6 Powstancow Warszawy, 35-959 Rzeszow, Poland; 2 Faculty of Medical Sciences, Rzeszow University of Information Technology and Management, 35-225 Rzeszow, Poland; 3 Department of Drug Technology and Biotechnology, Faculty of Chemistry, Warsaw University of Technology, 00-664 Warsaw, Poland; 4 Centre for Innovative Research in Medical and Natural Sciences, Faculty of Medicine, University of Rzeszow, 35-310 Rzeszow, Poland

**Keywords:** nimesulide, biotinylated PAMAM G3 dendrimer conjugates, normal human fibroblasts and squamous cell carcinoma, cytotoxicity, proliferation, COX-2/PGE_2_ axis

## Abstract

Squamous cell carcinoma (SCC) remains a main cause of mortality in patients with neck and head cancers, with poor prognosis and increased prevalence despite of available therapies. Recent studies have identified a role of cyclooxygenases, particularly inducible isoform cyclooxygenase-2 (COX-2) and its metabolite prostaglandin E2 (PGE_2_) in cancer cell proliferation, and its inhibition become a target for control of cancer development, particularly in the view of recognized additive or synergic action of COX-2 inhibitors with other forms of therapy. Nimesulide (N), the selective COX-2 inhibitor, inhibits growth and proliferation of various types of cancer cells by COX-2 dependent and independent mechanisms. In the presented study, the conjugates of biotinylated third generation poly(amidoamine) dendrimer (PAMAM) with covalently linked 18 (G3^B18N^) and 31 (G3^B31N^) nimesulide residues were synthesized and characterized by NMR spectroscopy. Biological properties of conjugates were evaluated, including cytotoxicity, proliferation, and caspase 3/7 activities in relation to COX-2/PGE_2_ axis signaling in human normal fibroblast (BJ) and squamous cell carcinoma (SCC-15). Both conjugates exerted a selective cytotoxicity against SCC-15 as compared with BJ cells at low 1.25–10 µM concentration range and their action in cancer cells was over 250-fold stronger than nimesulide alone. Conjugates overcome apoptosis resistance and sensitized SCC-15 cells to the apoptotic death independently of COX-2/PGE_2_ axis. In normal human fibroblasts the same concentrations of G3^B31N^ conjugate were less effective in inhibition of proliferation and induction of apoptosis, as measured by caspase 3/7 activity in a manner depending on increase of PGE_2_ production by either COX-1/COX-2.

## 1. Introduction

Squamous cell carcinoma (SCC-15) is a malignant tumor of squamous epithelium that may occur in many different organs, including skin. Oral squamous cell carcinoma (OSCC) is the most common malignant tumor of the oral cavity, accounting for over 90% of the malignant neoplasms in this location and is the sixth most common cancer in the world, with increasing incidences and five-year survival rate despite the available therapy [1,2]. Current treatment strategies for OSCC include surgery, chemo-, radio-, and photodynamic therapies often combined with epidermal growth factor receptor (EGFR) antagonists or cyclooxygenase-2 (COX-2) inhibitors, the last ones remaining under intensive elucidations [3].

Cyclooxygenases (COXs) are responsible for initiation of prostaglandin production cascade from membrane-derived arachidonic acid by its oxygenation to form prostaglandin G2 converted to prostaglandin H2 (PGH_2_) precursor of prostanoids, including PGE_2_ by the action of specific synthases and isomerases [4]. Three isoforms of COX are presently recognized: ubiquitously expressed constitutive COX-1, which is involved in homeostasis of vital physiological functions in most tissues, inducible COX-2 isoform, expressed during both inflammation and cancer, and COX-3, identified in human cerebral cortex and heart [5,6].

Many studies have identified the role of COXs in cancer development and progression [7]. Inducible COX-2 aroused particular interest since its expression was reported in various cancers, [8]. Thus, expression of COX-2 become a hallmark and diagnostic and prognostic index of cancer [9]. The use of specific COX-2 inhibitors became a target in control of various cancer type development, particularly in the view of their additive/synergic action with other forms of therapy [10,11,12,13,14,15].

Prostaglandin E2 is considered as the most important downstream cancer effector of COX-2. The elevated level of PGE_2_ was found in various cancer cells and it was correlated with high proliferation rate and reduced apoptosis [16]. The relationship between chronic COX-2/PGE_2_ mediated inflammation and development of many cancer types [17]. In various types of OSCC, up-regulation of COX-2 expression and high PGE_2_ level were reported, accompanied by increased proliferation rate, angiogenesis, and metastasis formation with inhibition of the apoptosis [4,18]. Development of various groups of specific COX-2 inhibitors and their pharmacological properties are described [19,20]. To the most considered and investigated selective COX-2 inhibitors belong the non-steroidal anti-inflammatory drugs (NSAID) of the coxibs group, and so-called preferential inhibitors including nimesulide [21,22]. Nimesulide (N) (n-(4-nitro-2-phenoxyphenyl) methane sulfonamide), the NSAID with sulfonanilide structure, is recognized as relative specific against COX-2 and is commonly used as an analgesic and anti-inflammatory agent in many countries of the world, excluding the United States, but it was approved by the European Medicines Agency in 2009. Symptoms of nephro- and hepatotoxicity of COX-2 inhibitors observed after prolonged treatment particularly in diabetic and kidney disease–suffering patients limits its application. However, the side effects accompanying the coxibs therapy (increased cardiovascular event risks) [23] are negligible when using nimesulide [22].

Growth inhibition and anti-proliferative action of nimesulide have been demonstrated against many cancer types in vitro and in vivo, including squamous cell carcinoma [24,25,26,27]. In order to decrease common side effects of multiple anti-cancer drugs, nanotechnology-based carriers, including polymeric nanoparticles and receptor mediated drug delivery nanosystems, are under investigations [3,28,29]. Proposed targeted therapy with N includes conjugates with various vehicles. Among them, significant activity was shown with poly(ethyleneglycol)-block-poly(ε-caprolactone) (PEG-b-PCL) particles against MCF-7 breast cancer cells [30]. Using hyaluronic acid as carrier, the inhibition of colorectal tumor growth and induced cell death via apoptosis was observed in vivo in nude mice, without symptoms of hepatotoxicity [31]. 

Dendrimers have been considered as promising candidates with their proper biocompatibility The cytotoxicity of the native dendrimers become an important issue, and many studies have undertaken this mater. It has been established that dendrimer cytotoxicity depends on size, structure, and chemical composition, in particular surface molecules and their chemical, biological, and electric properties, solubility and aggregation tendency [32]. Generally, cationic dendrimers of higher generations are more cytotoxic as compared with anionic or neutral small ones [33]. Thus, dendrimer surface substitution with various molecules (targeting, biologically active, or drugs) changes their biological properties, including cytotoxicity [34,35,36,37].

Other important observations concern very different dendrimer effects dependent on cell type and phenotype (normal or pathogenic, particularly cancer) cells [38]. Many experimental models investigated in vitro and in vivo documented that small dendrimers (up to fourth generation) may be useful as drug carriers [39]. Native G3 poly(amidoamine) dendrimer (PAMAM) revealed high loading ability and slow release of hydrophobic drugs, as compared to other dendrimer types, with low cytotoxicity against epithelial like cells from human breast carcinoma (MCF-7) and A549 human lung adenocarcinoma [40]. An increased tolerance for nimesulide delivery due to higher solubility and sustained release, as was described for third generation of quaternized poly(propyleneimine) dendrimer (QPPI G3) [41]. Moreover, targeted dendrimer–drug conjugates entering cells trough specific receptor endocytosis are better suited for specific delivery as compared with dendrimers loaded with the same drug, as was shown with PAMAM G5 conjugated with folic acid and methotrexate [42]. 

On the basis of our and other earlier studies, the focus was given to cationic PAMAM G3 amino terminated (-NH2) dendrimers that appeared to be good candidates for medical application after proper surface substitution [40,43,44,45].

The present aim was to investigate the PAMAM G3 dendrimer conjugated with biotin (B7 vitamin) as targeted carrier of nimesulide into SCC cells. Biotin has been recognized as targeting molecule into various tumor cells characterized by overexpression of biotin transporters (sodium multivitamin transporter: SMVT and monocarboxylate transporter: MCT-1) [43,46,47].

PAMAM G3 vehicle substituted with one amide-linked biotin (G3^B^) was synthesized according to optimized protocols [48,49] followed by covalent binding of N to obtain two conjugates containing average 18 and 31 residues of N (G3^B18N^ and G3^B31N^). The effects of both conjugates on cell viability, morphology, caspase 3/7 activity (as either pro-apoptotic or pro-cancer factor), COX-2 protein expression, and PGE_2_ production were investigated in human squamous cell carcinoma as compared to normal human fibroblasts in vitro. For comparison, the effects of equivalent concentrations of N alone were measured under the same conditions.

## 2. Materials and Methods

### 2.1. Materials

Solvents for chemical synthesis, nimesulide, trypsin-EDTA solution, phosphate-buffered saline (PBS) with and without magnesium and calcium ions, 0.33% neutral red solution, hydrocortisone, 0.4% trypan blue solution, protease inhibitor cocktail, and primary polyclonal anti-actin antibody were provided by Sigma–Aldrich (St Louis, MO, USA). Human normal fibroblasts (BJ), human squamous carcinoma (SCC-15), Eagle’s Minimum Essential Medium (EMEM), Dulbecco’s Modified Eagle’s Medium F12 (DMEM-F12), fetal bovine serum (FBS), penicillin, and streptomycin solution were obtained from American Type Culture Collection (ATCC, Manassas, VA, USA). Primary polyclonal anti-COX-2 antibody was obtained from Novus Biologicals (Centennial, CO, USA), and secondary conjugated with HRP polyclonal antibody was from Jackson Immuno Research (Cambridgeshire, UK). Reagents for electrophoresis and Western blotting were purchased in Bio-Rad (Bio-Rad Laboratories, Hercules, CA, USA). DAPI (4′,6-diamidino-2-phenylindole, dihydrochloride) and CellEvent™ Caspase-3/7 Green Detection Reagent were from Thermo Fisher Scientific (Waltham, MA, USA). Prostaglandin E2 Parameter Assay Kit was purchased from R&D Systems (Minneapolis, MN, USA). Cell culture dishes and other devices were from Corning Incorporated (Corning, NY, USA), Greiner (Austria), or Nunc (Denmark).

### 2.2. Synthesis

PAMAM G3 dendrimer was synthesized starting from ethylenediamine core by repeated alternate steps of Michael addition of methyl acrylate, followed by condensation of half-generation PAMAM dendrimer with ethylenediamine according to original protocol described by Tomalia for cystamine-core PAMAM dendrimers [48]. G3 was further substituted with single molecule of amide-bonded biotin by reaction of G3 with *N*-hydroxysuccinimido-biotin (NHS-biotin) as before to give monosubstituted G3^B^ [50].

The 21 mM solution of G3^B^ in dimethylsulfoxide (dmso) was further used to obtain conjugates with nimesulide (N). N was covalently attached to G3^B^ as follows: 0.4769 g of N (1.55 mmoles) was dissolved in 6.5 mL of chloroform and 300 µL of triethylamine was added. To this solution 0.3429 g (1.70 mmoles) of p-nitrophenylchloroformate (NPCF) was added in portions as solid with vigorous stirring at room temperature for 4 h. The product of substitution was purified by extraction in chloroform – water system, isolated from chloroform phase and purified chromatographically on silicagel with chloroform:ethyl acetate 7:1 *v*/*v*. The product was eluted as first fraction and identified by the ^1^H NMR spectroscopy (see Scheme 3 for atom numbering).

^1^H NMR (CDCl_3_): 8.25 (d, J_16-17_ = 9.2 Hz, [2H], H-17,19); 8.04 (dd, J_6-7_ = 8.7 Hz, J_6-4_ = 2.5 Hz, [1H], H-6); 7.70 (d, [1H], H-7); 7.69 (d, 1H], H-4); 7.46 (t, [1H], H-10,12); 7.32 (t, J_11-10_ = 7.3 Hz, [1H], H-11); 7.27 (d, [2H], H-16,20); 7.10 (d, J_9-10_ = 7.5 Hz, [2H], H-9,13); 3.54 (s, [3H], H-1). The resonance assignment based on COSY experiment.

Next, to a 1 mL of G3^B^ in dmso (21 µmoles) the solution of activated N was added 3.00 mL (synthesis 1, containing 684 µmoles of activated N) or 1.85 mL (synthesis 2; 410 µmoles of activated N). The mixtures were kept for 12 h, and then the solutions were dialyzed using nitrocellulose dialysis tube against water for four days. Solutions with precipitate were separated from dialysis tube, then solvents were removed under reduced pressure with rotary evaporator at temperature <50 °C and dried overnight at high vacuum (3 mm Hg).

The products were analyzed by ^1^H NMR spectroscopy in dmso-d_6_. For comparison, the ^1^H NMR spectrum of N in dmso was recorded and chemical shifts of ^1^H resonances assigned in accordance with literature data [51].

*^1^NMR (dmso-d_6_), chemical shift [ppm], multiplicity, intensity, assignment):*

N: 10.15, [1H], s, N-H; 8.03, [1H], dd, J_2,3_ = 9.1 Hz, J_3,5_ = 2.6 Hz, H-3; 7.73, [1H], d, H-2; 7.53, [1H], d, H-2; 7.48, [2H], t, J = 7.9 Hz, H-2′,5′; 7.27, [1H], t, J = 7.9 Hz, H-4′; 7.17, [2H], d, J = 7.9 Hz, H-2′,6′; 3.19, [3H], s, CH_3_-7.

G3^B18N^: 8.32 and 7.97, [18H], d and dd, H-3 (A) and H-3 (B); 7.61 and 7.44, [18H], d and d, H-2 (B) and H-2 (A); 7.52, [18H], bd, H-5 (A, B); 7.43, [36H], t, H-3′,5′ (A, B); 7.34 and 7.18, [18H], t and t, H-4′ (A) and H-4′ (B); 7.16 and 7.08, [36H], d and d, H-2′,6′ (A) and H-2′,6′ (B); 3.02, [54H], s, CH_3_-7 (A,B); PAMAM G3 broad resonances: 3.34, 3.09, 2.70, 2.22, [484H]; the broad NH (PAMAM) resonance is centered at 8.1 ppm; Biotin resonances: 1.64-1.21, [6H], -CH_2_-.

G3^B31N^: 8.32 and 8.01, [31H], d and dd, H-3 (A) and H-3 (B); 7.71 and 7.48, [31H], d and d, H-2 (B) and H-2 (A); 7.53, [31H], bd, H-5 (A, B); 7.37, [62H], t, H-4′ (A, B); 7.34 and 7.24, [31H], t and t, H-4′ (A) and H-4′ (B); 7.23 and 7.15, [62H], d and d, H-2′,6′ (A) and H-2′,6′ (B); 8.12 and 7.59, [31H], bs, NH (A,B); 3.15, [93H], s, CH_3_-7 (A,B); PAMAM G3 broad resonances: 3.5-2.1, [484H]; Biotin resonances: 1.64-1.21, [6H], -CH_2_-.

### 2.3. Cell Cultures

Human normal skin fibroblasts BJ (CRL-2522, ATCC) and squamous carcinoma cells SCC-15 (CRL-1623, ATCC) were cultured in EMEM and DMEM/F-12, respectively. Media were supplemented with 10% FBS and 100 U/mL penicillin/100 μg/mL streptomycin solution, and hydrocortisone (400 ng/mL) for carcinoma. Cells were cultured at 37 °C in humidified 95% air with 5% CO_2_, with changing media every 2–3 days and passaged at 70–80% confluence after trypsinization (0.25% trypsin-EDTA in PBS, calcium and magnesium free). The number and viability of cells were estimated by trypan blue exclusion test using Automatic Cell Counter TC20TM (Bio-Rad Laboratories, Hercules, CA, USA). All assays were performed as triplicates of three independent experiments. Cell morphology was observed under Nikon TE2000S Inverted Microscope (Tokyo, Japan) with phase contrast after treatment.

### 2.4. Cytotoxicity

Cells were seeded in flat-bottom 96-well plates at a density of 1 × 10^4^ cells/well (BJ) or 2 × 10^4^ (SCC-15) and allowed to attach for 24 h. Working solutions of dendrimers (0.625–10 µM) or N (25–800 µM) were prepared in culture media. The DMSO concentration were 0.4% (dendrimer conjugates) or 0.2% (N), that had no significant effect on treated cell lines. After 24 h exposure to dendrimers or N alone, the neutral red (NR) assay was performed as described [52].

### 2.5. Proliferation Assay

Cells were seeded in flat-bottom 96-well plates at a density of 5 × 10^3^ cells/well (BJ) or 1 × 10^4^ (SCC-15) and, after 24 h, treated with either dendrimer conjugate at 0.625–5 µM concentration range for 96 h. After removing the medium, cells were washed with PBS and fixed with 3.7% formaldehyde in PBS for 10 min. at room temperature (RT). Subsequently, nuclear DNA was labeled with 600 nM DAPI (30 min., RT, in darkness) and fluorescence was measured at 360/460 nm with Infinite M200 PRO Multimode Microplate Reader (TECAN Group Ltd., Switzerland). Results are triplicates of three independent experiments.

### 2.6. Apoptosis

Apoptosis was estimated with CellEvent™ Caspase-3/7 Green Detection Reagent (Thermo Fisher Scientific) assay as executive caspases 3 and 7 activity. Cells were seeded in flat-bottom solid black 96-well microplates (2 × 10^4^ or 4 × 10^4^ per well, BJ and SCC-15, respectively) and treated with 1.25–10 µM concentration range of dendrimer conjugates for 24 h. Working solution of CellEvent™ Caspase-3/7 Green Detection Reagent was added (4µ/well) and incubated (1 h, 37 °C). Fluorescence was measured at 490/530 nm with Tecan Infinite M200 PRO Multimode Microplate Reader (TECAN Group Ltd., Switzerland). Results from triplicates of three independent experiments were presented as a percentage of non-treated control.

### 2.7. Western Blot

Western blot analysis of the COX-2 protein level in cell lysates were prepared as described [53].

### 2.8. Prostaglandin E2 Production

Cells were seeded in a 24-well flat-bottom plates at density of 1 × 10^5^ cells/well (BJ) or 2 × 10^5^ (SCC-15). After 24 h cells were treated with conjugates at 1.25–10 µM concentration range for 24 h. Medium was removed, centrifuged (2000 r.p.m. 5 min. 4 °C) and frozen at −20 °C before assay. ELISA assay was performed according to manufacturer protocol [54].

### 2.9. Statistical Analysis

To estimate differences between G3^B18N^, G3^B31N^, PAMAM G3 and N treated and non-treated samples the statistical analysis was performed using non-parametric Kruskal–Wallis test or Mann-Whitney U test to estimate differences between G3^B18N^ and G3^B31N^ in appropriate concentrations for the same cell line. *p* < 0.05 was considered as statistically significant. Calculations were performed using Statistica PL 12.5 version software (StatSoft).

## 3. Results and Discussion

### 3.1. Bioconjugate Synthesis

*p*-Nitrophenylchloroformate (NPCF), the convenient reagent to provide one-atom linkers has been used to couple PEG with anticancer drug hydroxycamptothecin (HPTC) by formation of active carbonate with hydroxyl groups of PEG or HPTC [55] and conjugate PEG with PAMAM G5 and G6 dendrimers to construct effective non-viral gene delivery carriers [56]. Similarly, the hydroxyl group of block copolymer (BE) obtained from 1,2-butylene oxide and ethylene oxide were activated with NPCF to give NP-carbonate-BE [57]. The p-nitrophenyl substituent of the latter was then leaving group when reacted with amine group-ended PAMAM dendrimer to give linear-dendritic copolymer linked by amide. 

**Scheme 1:**
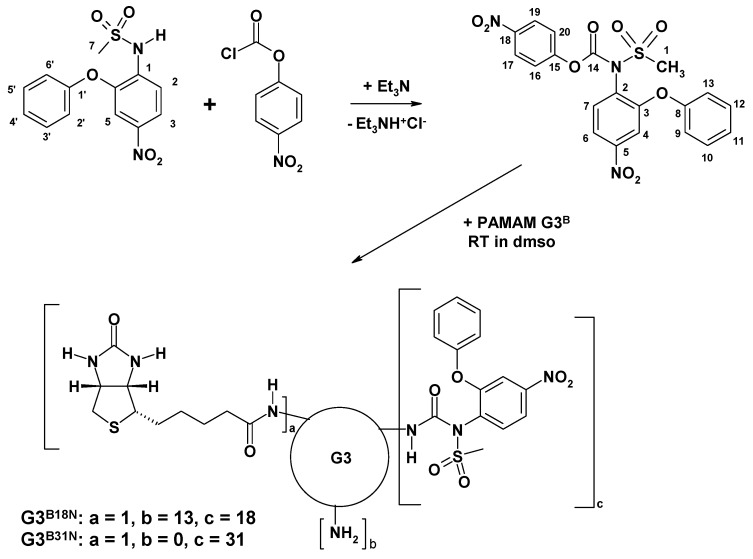
Synthesis of G3^B18N^ and G3^B31N^ conjugates.

Nimesulide (N) was demonstrated to react also with acyl chlorides, like acetate- or benzoil chlorides at ambient temperature, to obtain corresponding N-acetylated sulfonamide derivatives within couple of hours with > 70% yield [58]. We have applied NPCF to activate N by formation of N-(p-nitrophenylcarbonate)nimesulide, which was further used to conjugate N into PAMAM G3 dendrimer via carbonyl linker (Scheme 1).

**Figure 1:**
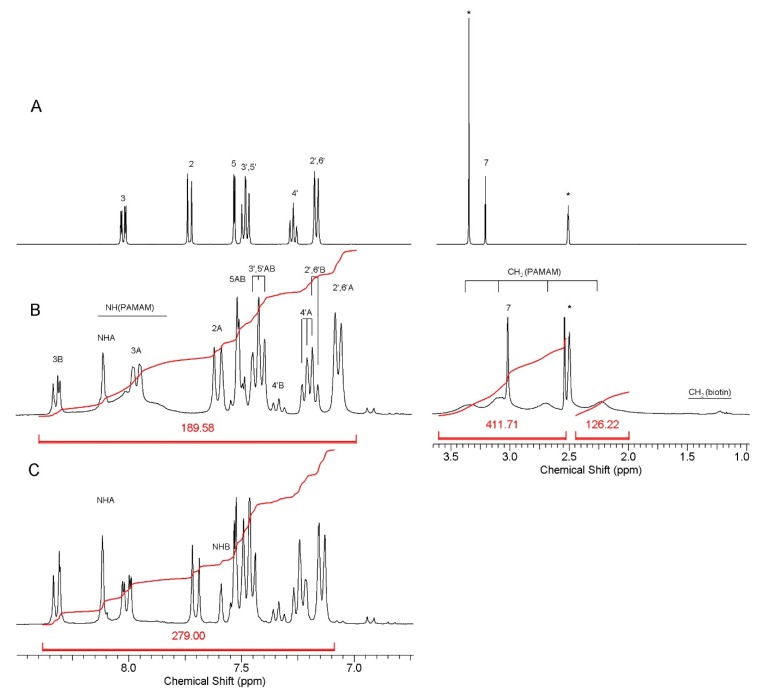
Relevant regions of ^1^H NMR spectra in dmso-*d*_6_ of: A—nimesulide, B—G3^B18N^, C—G3^B31N^. For numbering of N resonances, see Scheme 1. The N resonances of two isomers of carbonate amide linked N were combined into A and B sets due to scalar coupling observed in the ^1^H-^1^H COSY experiment performed for G3^B31N^. Amide linker NH proton resonances were identified by selective deuteration with D_2_O. Residual water and solvent resonances are labeled with asterisks. The intensity of resonances was divided by factor of four at the right side of spectrum B in order to fit the spectrum in window. For atom numbering of N, see Scheme 1. The residual *H*DO and CD_2_*H*SOCD_3_ resonances are labeled with asterisks.

The conjugates were evaluated by ^1^H NMR spectroscopy. Extensive dialysis of dimethylsulfoxide solutions of conjugates with water enabled to remove low molecular side products, like *p*-nitrophenol which was released at the final stage of synthesis. The products were better soluble in DMSO than in water. Based upon integral intensity of ^1^H resonances in the spectra of nimesulide containing conjugates the average number of 18 and 31 moieties of N, and an average of biotin was present in G3^B18N^ and G3^B31N^, respectively, as determined by ^1^H NMR. The NMR spectra of N and G3^B18N^, and G3^B31N^ conjugates are presented at Figure 1.

### 3.2. Cytotoxicity of G3^B18N^ and G3^B31N^ Conjugates as Compared with Nimesulide Alone and PAMAM G3

**Figure 2:**
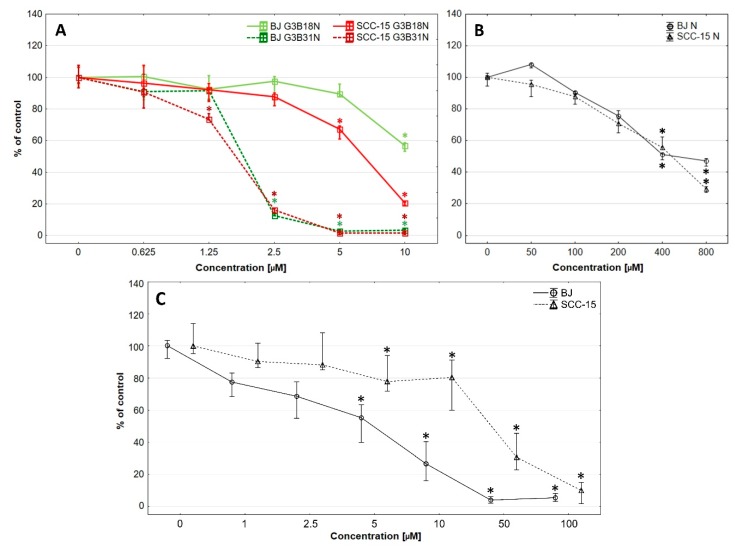
Cytotoxicity of G3^B18N^ and G3^B31N^ (**A**), nimesulide alone (**B**), or native PAMAM G3 dendrimer (**C**) against BJ and SCC-15 cells after 24 h incubation, expressed as percent of control. Results are expressed as percentage of non-treated control. Squares show medians and whiskers are lower and upper quartile. * *p* < 0.05 significant difference against control estimated with Kruskal-Wallis test.

Cytotoxicity estimated by NR assay revealed that both investigated cell lines were sensitive for either conjugate in concentration dependent manner (Figure 2).

The PAMAM cytotoxicity is influenced by generation, surface chemistry and dosage. The cytotoxicity of cationic PAMAM dendrimers is attributed to the interaction of surface cationic charge with negatively charged biological membranes that results in membrane damage via disruption of membrane structure and nanohole formation [59]. Many extensive studies have been performed in vitro using various models including lipid bilayers, liposomes, and Langmuir monolayers to study PAMAM dendrimer-membrane interactions [60,61,62]. It has been shown that low generation (<G5) of amine-terminated PAMAM dendrimers intercalate or adsorb to membrane surfaces rather than remove lipids. They are flexible and flatten against the membrane increasing the number of charge-charge interactions [63].

Review of PAMAM dendrimer toxicity and surface modifications for its reduction is given by Janaszewska et al. [37]. In general, cationic dendrimers were cytotoxic (72 h incubation), displaying IC50 values = 50–300 μg/mL dependent on dendrimer-type, cell-type and generation [64]. In vitro investigations of the cytotoxicity of native G3 dendrimers revealed that it differs very much depending on cell type. Well recognized is high neurotoxicity of cationic PAMAM dendrimers. G4 PAMAM with unmodified positively charged surface significantly reduced hippocampal neurons viability at 1 µM concentration [65]. G3 PAMAM affected human neural progenitor cell viability and neuronal differentiation at 10 µg/mL concentration [66]. Introduced chemical modifications has been shown to reduce of PAMAM dendrimer neurotoxicity [65,66,67]. 

Published data concerning the low generation PAMAM cationic dendrimers cytotoxicity for cancer cell lines amounted to vary different values with IC_50_ equal to 402 μM for human hepatocellular carcinoma (HepG2), 13.24 μM for human prostate cancer (DU145), 35 µM for murine melanoma cells (B16F10) [64,68]. PAMAM G3 were non-toxic at 20 µM concentration for human breast cancer (MCF-7) and at 60 µM for epithelial lung carcinoma (A549) cell lines [40]. Our earlier investigations of IC_50_ for native cationic PAMAM G3 reveal value 12.68 µM for SCC-15 cell line [49]. Less data are available for cationic PAMAM low generation cytotoxicity estimations against non-transfected cells. In human neural progenitor cells, a 10 μg/mL concentration significantly inhibited cell viability [66]. G4 dendrimers significantly reduced hippocampal neurons viability at 1 µM [65]. In our earlier studies, IC_50_ of G3 PAMAM for normal BJ fibroblasts was equal to 5.64 µM.

This diversity is due to complexity of mechanisms responsible for dendrimer cytotoxicity. The advanced studies on that issue, considering the neurotoxicity of higher generations (>4) of cationic PAMAM dendrimers, has been published and reviewed. This include apoptosis, mitochondrial activity, neuronal differentiation and gene expression due to oxidative stress and DNA damage [66,69]. Similar observations have been made for human colon cell line (SW480) and immortalized keratinocytes (HaCaT) with much higher sensitivity of HaCaT cells [70,71]. Wide range of the PAMAM G3 dendrimer cytotoxicity observed in various types of cells reveal the problem of its individual evaluation, depending on potential therapeutic target.

**Figure 3:**
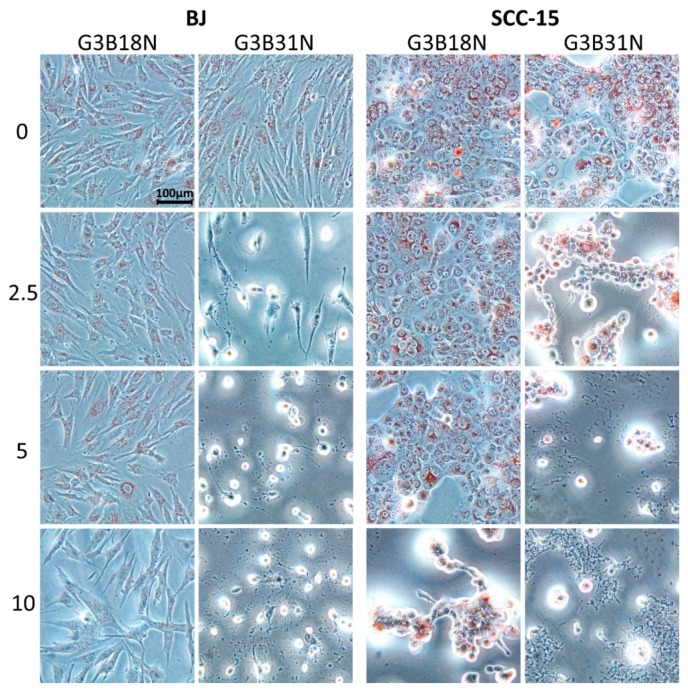
Morphology of BJ and SCC-15 cells after 24 h treatment with G3^B18N^ or G3^B31N^ as estimated with neutral red (NR) assay. Numbers indicate concentration (μM) of bioconjugates, red signal display the lysosomal NR accumulation in treated cells.

G3^B18N^ conjugate was significantly cytotoxic against SCC-15 cells at 5 µM concentration and against BJ cells at 10 µM concentration (about 70% and 55% of cell viability, respectively). It has to be pointed out, that at 10 μM G3^B18N^ concentration was nearly three times more toxic for cancer (20% viability) than fibroblasts (55% viability). G3^B31N^ conjugate with higher amount of N decreased remarkably SCC-15 cells viability at 1.25 µM and BJ cells at 2.5 µM concentration to 15% of control. At higher concentrations of G3^B31N^, no viable cells were seen. These results were confirmed by corresponding morphological changes observed in cultured cells (Figure 3). G3^B18N^ caused shrinking and aggregation of SCC-15 cells with reduction of NR accumulation at 10 µM concentration, whereas only small depletion of the dye accumulation was observed in BJ cells without loss of adhesion and noticeable changes in morphology. Severe morphology disturbances were visible in both cell lines treated with G3^B31N^ at 2.5 µM and dying cell morphology including fragmentation, degradation and loss of cell adhesion were seen at higher conjugate concentrations.

To compare the biological effects of the investigated nimesulide conjugated PAMAM dendrimers with N alone, the cytotoxicity assay with NR was performed (Figure 2B). The obtained results showed that the G3^BN18^ was over 295-fold, and G3^B31N^ was 266-fold more cytotoxic for the SCC-15 cells as compared to N alone (Figure 2A,B) In BJ cells, the differences were over 40- and 70-fold for G3^BN18^ and G3^B31N^, respectively. Calculated selectivity index (ratio of IC_50_ of cancer to IC_50_ of normal cells) revealed that dendrimer conjugates, particularly G3^BN18^, were more selective against cancer cells than N alone, with a selectivity index equal to 0.41 (Table 1).

Nimesulide cytotoxicity against cancer cells described by other investigators was considered as rather low. Estimated IC_50_ for SGC-7901 and AGC human gastric cancer cell lines amounted to about 400 µM (24 h incubation) and 250 µM (48 h incubation), respectively [72,73] and 136 μM (72 h incubation) for epidermal carcinoma KB cells (derived from OSCC) [74]. Our results obtained with N alone shown IC_50_ = 590 μM for BJ and 427 for SCC-15 cells, with SI = 0.72 (Table 1.). Conjugation of N with biotinylated PAMAM G3 remarkably increased activity of this drug compared to its native form. Estimated IC_50_ for SCC-15 cells were about 6.0 μM and 1.6 μM and for BJ cells 14.5 μM and 2.0 μM (G3^B18N^ and G3^B31N^, respectively). Moreover, our earlier study revealed that native PAMAM G3 dendrimer exhibited higher toxicity against normal fibroblasts (BJ) than squamous carcinoma cells (SCC-15) IC_50_ value estimated with NR assay after 24 h incubation was equal 5.64 µM for BJ cells and 12.68 µM for SCC-15 with selectivity index 2.25 [49]. G3 PAMAM dendrimer substituted with one biotin molecule showed similar cytotoxicity than native PAMAM G3 dendrimer (no significant differences). Thus, the biotinylation and substitution of PAMAM G3 with 18 or 31 residues of N changed its cytotoxicity profile and selectivity. This resulted in a higher cytotoxicity of G3^B18N^ against SCC-15 cells as compared to normal fibroblasts, with SI equal to 0.41 (Table 1).

### 3.3. Antiproliferative Activity

Antineoplastic agents kill cancer cells with a high rate of proliferation. However, this therapy also affects normal, rapidly dividing cells of the skin, hair follicles, or digestive tract, causing undesirable and damaging side effects [75]. Therefore, it is a matter of importance to compare the effect of investigated anti-cancer drugs with both cancer and normal human cells. 

**Figure 4:**
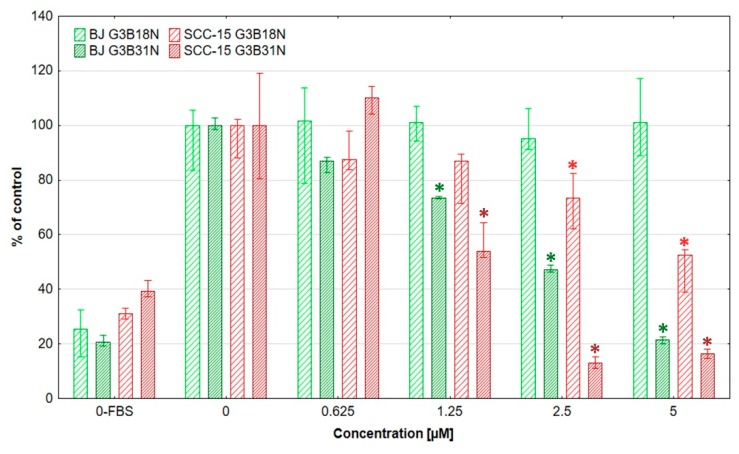
Proliferation rate of normal fibroblasts (BJ) and SCC-15 cells after 96 h incubation with dendrimer conjugates expressed as a percentage of non-treated control. 0-FBS indicate number of non-proliferating cells (deprived of FBS) as negative control. Bars are medians, and whiskers estimate lower and upper quartile values. * *p* < 0.05 indicate significant difference against control.

After 96 h incubation, both studied bioconjugates indicated anti proliferative action selective against SCC-15 cells, as estimated by fluorescence assay of nuclear DNA content with DAPI dye (Figure 4).

In proliferation assay the G3^B18N^ exerted high selectivity against cancer cells. Significant inhibition of proliferation of SCC-15 cells was seen at 2.5 µM concentration with decrease of cell number to about 30% and at 5 µM to about 50% of control, with no significant changes noticed in normal fibroblasts. G3^B31N^ inhibited proliferation at 1.25 µM concentration for both SCC-15 and BJ cells. At 2.5 μM concentration, also cytotoxic effect was seen in SCC-15 cells (number of cells was below of that in 0-FBS culture) and antiproliferative action against fibroblasts (about 50% of control).

Antiproliferative properties of N against various types of cancer cells were observed by the others, but estimated drug concentrations were rather high, with IC_50_ in range of 30-800 µM [24,76]. N conjugated with hyaluronic acid decreased proliferation of colorectal cancer cells (HT-29, HCT-15) with IC_50_ about 400 µM as compared to 1600 µM for N alone [31]. Hida et al. have shown the selective antitumor action of N against non-small cell lung cancer as compared with normal human epithelial cells and poor response to N treatment of the human squamous cell lung carcinoma–derived PC-10 cells. In OSCC KB cells the N treatment at 100 µM for 72 h resulted in 43.3% of proliferation inhibition, with estimated IC_50_ equal to 130 µM. However, no induction of DNA fragmentation at nimesulide concentrations of up to 200 µM after 24 h incubation was observed [76]. In two head-and-neck carcinoma cell lines (SCC9 and SCC25), N (50–600 μM) inhibited cell proliferation without affecting colony-forming ability [10].

The highly potentiated effect of nimesulide conjugated with biotinylated PAMAM G3 dendrimer as inhibitor of proliferation of SCC-15 cells is clearly visible. It has to be considered that cancer cell growth and proliferation may be affected by local interactions of dendrimer conjugates surface molecules with cell membranes, particularly with receptors of cell surviving and death signals acting as stimulants or inhibitors. This would depend on dendrimer size, surface molecule charge, reactivity, and concentration. For small nanoparticles (<10 nm), it has been found that rare-earth fluoride nanoparticles at low concentration stimulate proliferation of three human cell lines—A549, SW837, and MCF-7—through activation of EGFR and integrin signaling [77]. Native PAMAM G3 dendrimer molecule has about 3.2 nm diameter. At higher concentration of the conjugates, the inhibitory effect may appear. Currently, a new concept of cell fate regulation including interaction with adhesion and extracellular matrix-based mechanisms and new mechanisms of mechanotransduction has been proposed that may explain this experimental data [78,79].

### 3.4. Apoptosis

**Figure 5:**
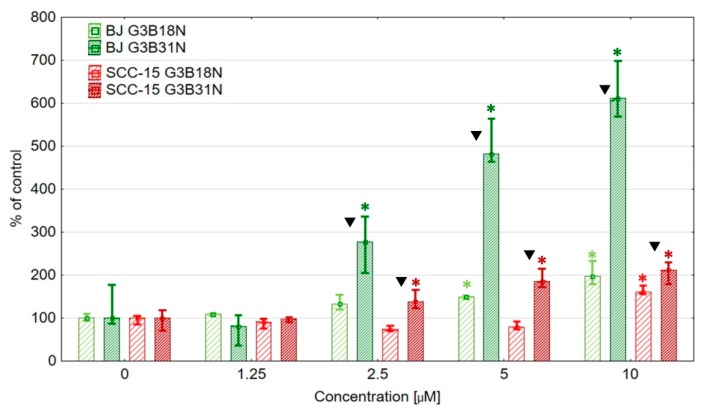
Activity of executive 3/7 caspases after 24 h treatment of BJ and SCC-15 cells with G3^B18N^ and G3^B31N^ conjugates, expressed as a percentage of control. Squares show medians, and whiskers are lower and upper quartile. * *p* < 0.05 significant difference against control (Kruskal-Wallis test). Triangles show significant differences between conjugates for the same cell lines (Mann Whitney U test, *p* < 0.05).

Inhibition of apoptosis is the canonical marker of cancer and has been a target for the development of anticancer strategies. Activation of the specific cysteine proteases (caspases) plays a central role in the cascade reactions realizing apoptotic program [80]. The ability of biotinylated PAMAM G3 dendrimers conjugated with N to induction of apoptosis was evaluated by measuring the activity of executive caspases 3 and 7 (Figure 5). Both dendrimer conjugates revealed concentration dependent, pro-apoptotic effect with higher caspase 3/7 activity seen in fibroblasts. Significant G3^B18N^ stimulatory effect was seen at 5–10 µM concentrations. At 10 µM concentration, caspase activity was doubled in BJ and increased by 60% in SCC-15 cells (Figure 5). 

The effect of G3^B31N^ conjugate, carrying the higher load of N, was much more pronounced, particularly for fibroblasts. In BJ cells, an increase of caspase activity was observed at 2.5 µM concentration (about three-fold of control), with further increase to 5–6 fold of control at 5–10 µM concentrations, respectively (Figure 5). A smaller but significant effect was seen in SCC-15 cells. At 2.5 µM concentration, an increase of caspase activity amounted to 150%, and at higher concentrations, it was doubled, as compared with control (Figure 5). 

Induction of apoptosis by N has been recognized in many types of cancer cells including human pancreatic cancer (PANC-1), lung cancer (A549), gastric cancer (SGC-7901), and squamous carcinoma cells (KB, SCC9, SCC15) at rather high concentrations (in the range of 100–400 µM) [13,26,81]. In OSCC KB cells, N at 100 μM concentration was not able to induce apoptosis as detected by the poly(ADP-ribose) polymerase (PARP) fragmentation and DNA fragmentation assays, and up to 200 μM did not alter the basal level of caspase-3/7 activity [74]. Both dendrimer conjugates indicated significant induction of apoptosis measured as activity of caspases 3/7 at 2.5–10 µM concentrations with much higher effect in fibroblasts. However, a matter of consideration is validation of the use of executive caspase 3/7 assay as measure of apoptosis [82]. Executive 3/7 caspases have been recognized as executioners of apoptosis, which made them a target in anti-cancer therapy, but their function in proper organism development, tissue homeostasis, and post-injury recovery has to be remembered. Many investigations revealed non-apoptotic functions of that enzymes [83]. New evidence reveals that activation of caspases in many cancers plays a pivotal role in maintaining their tumorigenicity and metastasis [84,85,86]. Particularly important is a phenomenon termed apoptosis-induced proliferation (AiP) causing proliferation cells by apoptotic caspases activated in stress-induced dying cells and its role in tissue regeneration and cancerogenesis [87,88,89]. The obtained results reveal activation of caspases 3/7 by tested dendrimer conjugates at low 2.5–10 μM concentrations in both fibroblasts (to higher degree) and squamous carcinoma cell line (to lower degree). However, mechanisms of that phenomenon seem to be different. Smaller inhibitory effect on BJ cell viability and proliferation and higher stimulation of caspase 3/7 activity may be due to AiP effect. In SCC-15 opposite effects were seen that may be result of prevalence of pro-apoptotic effect of N dendrimer conjugates. More information concerning the mechanisms of OSCC cell death induced by investigated conjugates nimesulide requires investigations of other death pathways including autophagy and necrosis (for review, see [90]).

### 3.5. COX-2 Expression and Prostaglandin E2 Production

In order to evaluate the role of COX-2/PGE_2_ axis in anticancer properties of investigated conjugates the COX-2 expression and prostaglandin E2 production were estimated. Analysis of COX-2 protein expression revealed two bands recognized by specific anti-COX-2 antibody, similar to that seen by the others with OSCC samples, and is due to different glycosylation profile of COX-2 protein [91,92]. The basal level of COX-2 protein was found in normal human proliferating fibroblasts [53,93]. Animal studies reveal that COX-2 is constitutively expressed in various tissues and organs (the renal medulla and pelvis, the gastrointestinal tract, lung, thymus, and brain) in the absence of inflammation signaling pathways [94]. 

**Figure 6:**
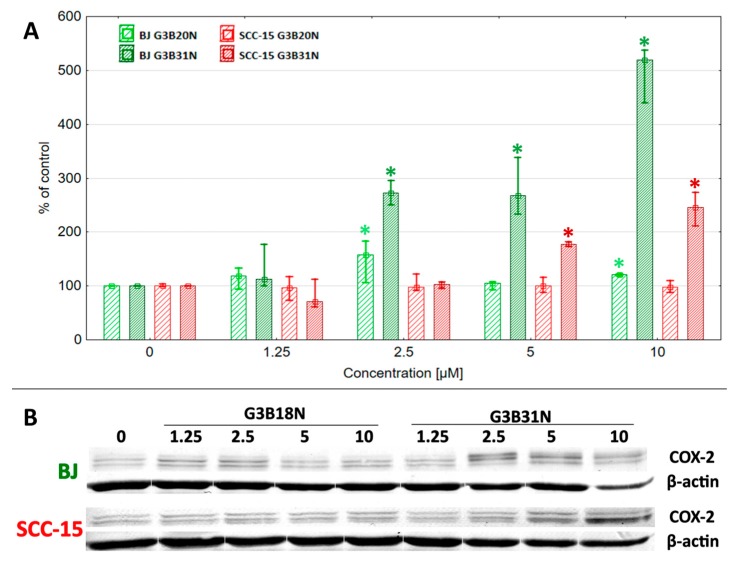
Effect of G3^BN18^ and G3^BN31^ on expression of COX-2 protein in human normal fibroblasts (BJ) and squamous carcinoma cells (SCC-15). (**A**) Levels of COX-2 protein calculated against β-actin were presented as the percent of the control. Squares indicates medians and whiskers correspond to the lower (25%) and upper (75%) quartile ranges. * *p* < 0.05 significant difference against control (Kruskal–Wallis test). (**B**) Representative images of Western blot.

As was mentioned in the Introduction, high expression of COX-2 protein is a marker of a majority of malignances. Immunohistochemical studies of the expression of COX-2 in human oral squamous cell carcinoma (OSCC), premalignant lesions, and normal oral epithelium reveal the high level of COX-2 protein in OSCC and dysplasia compared to normal mucosa [91,95]. No effect of G3^BN18^ treatment on COX-2 protein level was observed in SCC-15 cells at all tested concentrations, whereas G3^BN31^ significantly induced enzyme expression at 5–10 μM concentrations to about twice the control level (Figure 6). In fibroblasts, G3^BN18^ exerted limited effect on COX-2 expression with an increase of up to 170% of control at 2.5 μM concentration and without/small effect (120%) at 5–10 μM concentration. More remarkable changes were observed after G3^BN31^ treatment that induced of COX-2 protein expression, up to three- and five-fold of control level at 2.5–10 μM concentration (Figure 6).

The stimulation of COX-2 expression by its inhibitors was reported by the others. Ko at al. observed that four COX-2 inhibitors, celecoxib, NS-398, nimesulide, and meloxicam increased COX-2 protein expression in the OCSC KB cells [74]. Nimesulide treatment (100 µM for 24 h) doubled the level of COX-2 protein. Similarly, stimulated expression of COX-2 was seen at 72-h treatment with 100 μM NS398 in OSCC cancer samples and cell lines [91]. Induction of enzyme protein expression by its inhibitors is a recognized phenomenon in cancer cells. The increase of dihydrofolate reductase and thymidylate synthase protein expression is considered to be part of the mechanism of methotrexate and 5FU resistance in human lymphoma cells [96,97,98].

**Figure 7:**
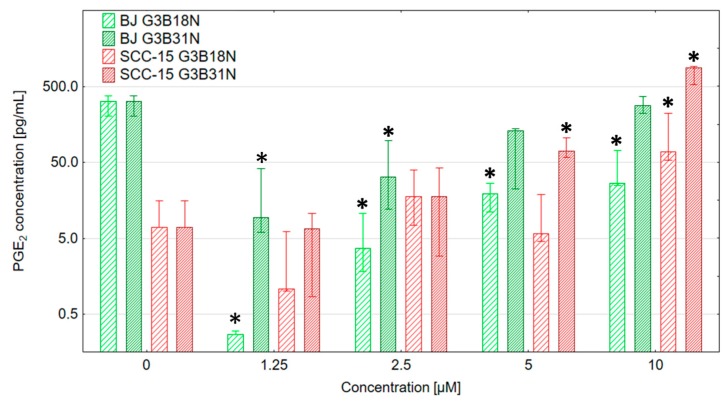
PGE_2_ production by BJ and SCC-15 cells treated with G3^B18N^ or G3^B31N^ for 24 h estimated by ELISA. Bars show medians, whiskers are lower and upper quartile. * *p* < 0.05 significant difference against control (Kruskal-Wallis test).

Estimation of PGE_2_ production and the effect of dendrimer conjugates reveal a different pattern in investigated cell lines. In fibroblasts, both G3^BN18^ and G3^BN31^ significantly decreased PGE_2_ production at 1.25 µM concentration, which confirmed the inhibitory action of dendrimer-bound nimesulide on COX-2 activity. At higher concentrations, both conjugate effects become smaller, and the level of PGE_2_ production, although lower than in control, become non-significant. This allows us to consider that nimesulide conjugates in BJ cells decrease PGE_2_ production despite a significant increase of COX 2 protein expression (Figure 6 and Figure 7).

In SCC-15 cells, the basal level of PGE_2_ was very low, and dendrimer conjugate treatment was without effect up to a 2.5 µM concentration. However, increased PGE_2_ production was observed at higher concentrations—at 5 µM with G3^BN31^ and at 10 µM with both conjugates. That was parallel with a significant increase of COX-2 protein level (Figure 6 and Figure 7). This is in contrary to observations of Ko et al., who found that at concentration range 0.5–50 μM the COX-2 inhibitors (celecoxib, NS-398, meloxicam, and nimesulide) significantly suppressed PGE_2_ production. Nimesulide after 1 h treatment at 0.5 μM concentration decreased PGE_2_ production to 20% of control in OSCC KB cells [74]. COX-2-selective inhibitor NS398 dramatically reduced PGE_2_ secretion in oral carcinoma-derived cell lines at 10 and 20 μM concentrations, but growth inhibition was not observed after 72 h treatment [91].

The complexity of the COX-2 effect on cancer cell death was revealed by studies of Totzke et al. Nimesulide effects were investigated in TNF-resistant HeLa H21 and TNF-sensitive HeLa D98 cells and MCF-7 cells transfected with COX-2. Nimesulide sensitized cancer cells to extrinsic death receptor-induced apoptotic pathway (TNF, CD95, and TRAIL receptors), independently of COX-2 presence and PGE_2_ production. The phenomenon that the COX-2 expression may be not accompanied by COX-2 activity and PGE_2_ secretion is supported by many investigations, and various mechanisms are recognized. This includes COX-2 itself and its inhibitors effects on various signaling pathways in normal and cancer cells, including inhibition of various apoptotic signals or modulation of cell cycle checkpoints [15,99,100,101,102]. Particularly interesting are observations that various selective COX-2 inhibitors decrease the proliferation rate and induce apoptosis in a manner not related to the COX-2/PGE_2_ axis [103].

The phenomenon observed in SCC-15 cells that, despite the inhibitory action of G3^B18N^ and particularly G3^B31N^ conjugates on proliferation rate at low concentrations (1.25–5 μM), only a small increase of caspase 3/7 activity and no increase of PGE_2_ production was seen, is in agreement with other data, showing that much lower concentrations of N affects the death of cancer cells than that required for COX-2 inhibition [74]. Thus, it allowed us to assume that nimesulide-conjugated dendrimers executed their anti-cancer activity mainly by COX-2/PGE_2_ independent signaling pathways.

Inhibitory effect of conjugates on PGE_2_ production in fibroblasts, despite the increased caspase 3/7 activity and high COX-2 protein expression, may indicate that at low concentrations dendrimer conjugates inhibit COX-2 activity. However, the G3^B31N^, with higher amount of nimesulide, exerted a much lower inhibitory effect up to a 2.5 µM concentration and was without effect at 5–10 µM. This may be explained by recent investigations concerning the functional interchangeability of two COX isoforms. Inhibition of COX-2 by coxibs, beside the inhibition of COX-2-mediated inflammatory prostaglandins causes also shifts prostanoids synthesis toward the COX-1-mediated pathway in human arthritis cartilage [104]. The functional interchangeability of these two COX isoforms is also confirmed by [105] and [106] with an animal cell model. Similarly, recently published observations by [107] concerning genomic, lipidomic, and metabolomic analysis of wild-type and cyclooxygenase-null mice lung fibroblasts revealed increased COX-1 activity and production of PGE_2_ in COX-2^−/−^ cells and showed the compensation of various eicosanoids at the genomic and metabolic levels.

## 4. Conclusions

Substitution of biotinylated PAMAM G3 dendrimers with 18 (G3^B18N^) or 31 (G3^B31N^) nimesulide residues remarkably increased activity of this drug compared to its native form. IC_50_ estimated for G3^B18N^ was 6.0 μM and 14.5 μM in SCC-15 and BJ cells, respectively, with SI = 0.41 as compared to IC_50_ equal to 430 μM and 590 μM for nimesulide alone. Our data suggest that biotinylated dendrimer conjugated with nimesulide at low concentrations (1.25–10 µM) inhibits proliferation, overcomes apoptosis resistance, and selectively sensitizes squamous carcinoma cells SCC-15 to the apoptotic pathway independently of the COX-2/PGE_2_ axis. In normal human fibroblasts (BJ), the same concentrations of G3^B31N^ conjugate was less effective in inhibition of proliferation and increased apoptosis as measured by caspase 3/7 activity in a manner depending on the increase of PGE_2_ production by either COX-1/COX-2. In conclusion, the nimesulide dendrimer conjugates are potential candidates for local squamous cell carcinoma treatment, but it has to be taken into consideration that their effects are concentration- and cell type (normal/cancer)–dependent.

## Figures and Tables

**Table 1:** IC_50_ values and Selectivity Index (SI) obtained with NR assay after 24 h treatment with dendrimer conjugates, native PAMAM G3, or nimesulide alone.

Compound	IC_50_ [µM]		Selectivity Index (SI)
	BJ	SCC-15	
PAMAM G3	5.64	12.68	2.25
Nimesulide	587.30	426.90	0.72
G3^B18N^	14.50	5.96	0.41
G3^B31N^	1.99	1.60	0.80
